# From Mid-Level Policy Analysis to Macro-Level Political Economy: Comment on "Developing a Framework for a Program Theory-Based Approach to Evaluating Policy Processes and Outcomes: Health in All Policies in South Australia"

**DOI:** 10.15171/ijhpm.2018.12

**Published:** 2018-02-07

**Authors:** Ronald Labonté

**Affiliations:** Canada Research Chair, Globalization and Health Equity, Faculty of Medicine, School of Epidemiology and Public Health, University of Ottawa, Ottawa, ON, Canada.

**Keywords:** Health in All Policies, Evaluation Research, Program Theory, Political Economy

## Abstract

This latest contribution by the evaluation research team at Flinders University/Southgate Institute on their multiyear study of South Australia’s Health in All Policies (HiAP) initiative is simultaneously frustrating, exemplary, and partial. It is frustrating because it does not yet reveal the extent to which the initiative achieved its stated outcomes; that awaits further papers. It is exemplary in describing an evaluation research design in which the research team has excelled over the years, and in adding to it an element of theory testing and re-testing. It is partial, in that the political and economic context considered important in examining both process and outcome of the HiAP initiative stops at the Australian state’s borders as if the macro-level national and global political economy (and its power relations) have little or no bearing on the sustainability of the policy learning that the initiative may have engendered. To ask that of an otherwise elegant study design that effectively engages policy actors in its implementation may be demanding too much; but it may now be time that more critical political economy theories join with those that elaborate well the more routine praxis of public policy-making.

There are two ways in which one can approach the well-crafted paper by Lawless et al^1^: as a contribution to a deeper understanding of the potential for Health in All Policies (HiAP) to achieve notable health-positive outcomes, or as an approach to policy studies more generally. The two ways are not exclusive, although as a contribution to the literature on HiAP the paper is frustratingly limited, focusing as it does on an evaluation design rather than on outcomes *per se*. There is only passing reference to a single HiAP example (changes to improve bicycle paths, which the authors acknowledge still awaits findings on the more distal outcomes), with the teasing promise of future results-oriented papers based on interview and survey data and drawing from several detailed case studies. Process evaluations of these HiAP ‘health lens analysis’ [HLA] case studies have already been posted by the South Australian Health Department,^2^ including a summary report providing a rationale for their selection^3^; as well as earlier papers by the authors that argue that the HLA process ‘had a number of positive effects’^4^ (p.i138), and an analysis of enablers or barriers to the HiAP process.^5^ These assessments, however, remain descriptive evaluations of process and are not the ‘detailed findings…shaped by the framework’ in the present article^1^ (p.10) being promised in subsequent papers. Presumably these papers-in-progress will include analyses of changes in government investments and policies, and offer some assessment of whether South Australia has indeed become ‘a better place to live with increased population health and equity’ (two of the outcomes noted in Figure 1). In terms of whether or not HiAP leads to sustained, health equitable impacts, at least as manifest through the South Australian initiative, thus remains a matter of future anticipation.

It is the second reading of the paper, as a reasonably detailed summary of how one might pry open the ‘black box’ of policy processes such as those undertaken in the South Australian HiAP initiative, that the authors make a solid contribution to the public health literature. Adopting what appears to be a constructivist epistemology with its emphasis on the dynamic role of actors in meaning and knowledge construction,^6^ the paper begins by acknowledging many of the travails of contemporary policy research: from difficulties in getting access to policy makers, to the contingent dynamics of day-to-day politics (in French, *politique* means both policy and politics which in English has somehow become bifurcated concepts), to challenges in causal attribution. While acknowledging that the policy world is ‘complex’ (a term invoked seven times in the article), the theory-based approach they outline is surprisingly devoid of reference to complexity theory itself (Table 2). Since the evaluation design described in the article is based upon processual analyses and the co-creation of meaning (including the Evaluation Framework portrayed in Figure 1), the research team may have assumed that their study itself stands as an example of a complex adaptive system and therefore has no need to cite it as such. To the authors’ credit, they eschew the almost prerequisite nod in contemporary public health policy research to the concept of ‘wicked problems,’ a term that I have taken to define as those problems that arise from the deeply embedded contradictions of a hyperglobalized capitalism that analysists would prefer to bracket out of consideration.

Some of the elements of the study design outlined in the paper are fairly well-known within the extant corpus of program evaluation: Analyses of policy and program documents, use of program logic-models, their co-creation with government policy or program decision-making, triangulated use of nested case studies and interview and/or survey data, and an iterative approach that continually presents findings-in-process during critically reflective events, sometimes drawing in new commentators removed from the studies under review. It is an elegant design that the Flinders University/Southgate Institute research team has refined and excelled in over the years, having applied it in multiple other studies.^7,8^ (Full disclosure: I have been involved in some of these other studies with this team, albeit not the one under present consideration.) The group, I would contend, has established itself international leaders in such evaluation research, with a long and credible record of rigorous outputs and a deep commitment to working with government, indigenous, and community-sector actors. Figure 2 in the article presents a useful summation of their approach, while also introducing an important and somewhat novel element: that of theory-testing and re-testing, in which the implicit theorizing of policy and program practitioners as captured in a program logic model (the Evaluation Framework present as Figure 1) is enriched (or as the article promises, will eventually be enriched) by introducing analytical elements from multiple social and political science theories (Table 2).

With respect to the challenge of attribution, the core of their HiAP evaluation design rests upon ‘contribution analysis,’ in which the different theories that inform and/or derive from the program logic (the Evaluation Framework) can and will be used to construct a step-wise causal chain, similar to comparative historical analysis studies that have long been the methodological bread and butter of the social sciences.^9^ Once such pathways (logic models) have been constructed, multiple data methods can be used in triangulation to assess links along the chain, creating a rigorous causal narrative in place of attempts to establish causality between a single input (eg, a policy change) and a distal output (eg, an improvement in a health outcome). Such single cause/effect models, and the randomized controlled trials used to test them, may remain the gold standard in laboratory science but have never fit well with the ‘complexity’ of politically dynamic policy environments. Even the rare exceptions afforded by natural experiments (mimicking randomization) are limited in explanatory force due to the number of possible confounding variables. The strength of the contribution analysis proposed by the authors for their forthcoming detailed evaluations is that it should allow for nuanced explanation of the ‘how’ and ‘why’ of changes in the outcomes its results-oriented studies will (presumably) document. This, in turn, will allow for a robust testing of the program theory (the pathways of Figure 1 as enhanced by the theories elaborated in Table 2).

At the same time, there are some intriguing silences in the present paper, which may also speak to limitations of a HiAP approach itself. Although the paper gives considerable emphasis to the importance of context, there is a certain parochialism in where the boundaries have been drawn. Oblique reference is made to ‘political priorities of the day’ and ‘budgetary considerations,’ but the absence of considerations of political and economic shifts at the national or global scales (eg, hyperglobalization, capital mobility, economic financialization, and tax competition) assumes that the policy actors at the South Australian state level have the capacities to implement HiAP in ways that can institutionalize sustained improvements in health equity. There is no discussion or even acknowledgement of the deeper and more structurally embedded contextual factors that condition and constrain both the processes of HiAP, and its intended health equity outcomes. It is not that the authors are ignorant of these factors, given the extent to which that they have written about them elsewhere.^10^ But it leads to lacunae in the Evaluation Framework (a lack of national and global contextualization), the theories being invoked (whither macro-level political economy?), and, as one example, any explanation for why affiliated health promotion activities were abandoned by a government nominally supportive of HiAP. Having in the state at the time this occurred, I know that this decision was driven largely by cost-containment arguments built on a bad evidence review with a neoliberal ideological undertone, which rather begs the deeper question: why was the government faced with perceived fiscal scarcity requiring such a cost-containment review in the first place?

The authors are far from alone in side-stepping such macro-level interrogation. Much of my own work on HiAP or related themes similarly has focused more on the quotidian and mid-level dynamics of program and policy change than on the restraining impacts of a global political economy that continues to exacerbate wealth and income inequalities while pushing the planet closer to an ecological Armageddon. True, most of my present work (at least that which I now personally lead) orients more towards such globalization themes, a personal predilection that undoubtedly colours my commentary. But it also gives me pause to consider whether it is not incumbent now for all policy-relevant and action-oriented research on health equity to adopt a ‘global’ HiAP contextual frame, even if only as an introductory or concluding note. Assessing the impacts of the mid-level health-promoting policy and program change that this larger HiAP study will eventually complete remains an important task, but it may no longer be a sufficient one. I appreciate that this may be asking far more of individual research studies or peer-reviewed papers than is possible for them to accomplish. It might also be less likely when one’s co-creators in the research process are mid-level policy-makers themselves, since they may be less prone (or able) to consider analyses with such explicitly critical political theorizing. But I find it hard to accept that the political and ecological *zeitgeist* of our time demands anything less, which I offer less as a critique of the present paper than as an invitation for all public health researchers not only to pry deeper into the black-box of policy complexity, but to use the force of public health engagement and advocacy to force and keep open the ‘opportunity windows’ on what have become the two defining meta-health issues (inequality and sustainability) of our time.

Accepting this single *caveat*, and considering the present paper as an articulation of an approach to mid-level policy and program evaluation that combines (some) theory with (solid) practice alongside (very good) methodological rigour, the authors succeed admirably in providing activist researchers with a useful roadmap on how to begin to answer the all-important ‘so what?’ policy question, HiAP or otherwise. Given the small size of the South Australian state, any eventual conclusions the broader study finds will be somewhat limited in generalizability or transferability beyond its own political confines. The approach it takes to the evaluation study, however, stands as an important and solid contribution to the public health corpus on how ‘healthier public policies’ might move from idea to articulation to implementation.

## Ethical issues

Not applicable.

## Competing interests

Author declares that he has no competing interests.

## Author’s contribution

RL is the single author of the paper.
